# Coumestrol mitigates retinal cell inflammation, apoptosis, and oxidative stress in a rat model of diabetic retinopathy via activation of SIRT1

**DOI:** 10.18632/aging.202467

**Published:** 2021-02-01

**Authors:** Yanchao Xu, Yusong Zhang, Hongwei Liang, Xiaomeng Liu

**Affiliations:** 1 The Second Ward, Department of Endocrinology and Metabolism, Linyi People’s Hospital of Shandong Province, Linyi 276000, P. R. China; 2 Imaging Center, Linyi People’s Hospital of Shandong Province, Linyi 276000, P. R. China; 3 Department of Health Care, Linyi People’s Hospital of Shandong Province, Linyi 276000, P. R. China

**Keywords:** coumestrol, sirtuin 1, diabetic retinopathy, inflammation, apoptosis

## Abstract

Diabetes-induced oxidative stress is vital in initiating neuronal damage in the diabetic retina, leading to diabetic retinopathy (DR). This study investigates the possible effects of coumestrol (CMS) on streptozotocin (STZ)-induced DR. First, we established a rat model of DR by STZ injection and a cell model involving high-glucose (HG) exposure of human retinal microvascular endothelial cells (hRMECs). We characterized the expression patterns of oxidative stress indicators, pro-inflammatory cytokines, and pro-apoptotic proteins in hRMECs. Polymerase chain reaction showed sirtuin 1 (SIRT1) to be poorly expressed in the retinal tissues of STZ-treated rats and HG-exposed hRMECs, but its expression was upregulated upon treatment with CMS treatment. Furthermore, CMS treatment attenuated the STZ-induced pathologies such as oxidative stress, inflammation, and cell apoptosis. Consistent with the *in vivo* results, CMS activated the expression of SIRT1, thereby inhibiting oxidative stress, inflammation, and apoptosis of HG-treated hRMECs. From these findings, we concluded that CMS ameliorated DR by inhibiting inflammation, apoptosis and oxidative stress through activation of SIRT1.

## INTRODUCTION

Diabetic retinopathy (DR), which is a major microvascular complication of diabetes mellitus (DM), is regarded as the leading cause of visual impairment and blindness worldwide [1]. Statistics indicate DR as a cause of 2.4 million cases of blindness globally [2], accompanied by an annual incidence of 2.2% to 12.7% [3]. As a visual impairment disorder, DR is characterized by retinal neuronal and vascular dysfunction in the early stages, which often progresses to extensive impairment of visual acuity by neovascularization [4]. Angiogenesis, oxidative stress, as well as chronic inflammation, have been identified as crucial processes implicated in the pathogenesis of DR [5]. The main risk factors, including hyperglycaemia, hypertension, advanced age, insulin treatment, induced fasting blood glucose level, longer DM duration, higher haemoglobin A1c concentration, and diet, have all been ascertained to promote the occurrence and progression of DR [2, 6, 7]. Despite advances in improving the the retinal vascular alterations, DR still poses as a major challenge in clinical therapy [8]. Thus, fundamental molecular investigations are needed for the development of better treatment strategies against DR.

Phytoestrogens are plant metabolites that serve as modulators with important activities on vertebrate estrogen receptors, thereby affecting glucose homeostasis, and helping to prevent DM among women [9, 10]. For example, daidzein has been reported to be a potential candidate for the treatment of DM [11]. Coumestrol (CMS), a coumestan isoflavone, vitally functions in the treatment of estrogen-linked pathologies such as DM [12]. A previous study demonstrated the effectiveness of 10-hydroxy-CMS as an antihyperlipidemic agent in streptozotocin (STZ)-induced diabetes [13]. Furthermore, CMS could intrinsically stimulate mitochondrial biogenesis by activation of Sirtuin 1 (SIRT1) in skeletal muscle cells [14]. Specifically, a recent study highlighted that down-regulating the expression of microRNA-377 inhibited high glucose (HG) and hypoxia-induced angiogenic effects and suppressed the release of pro-inflammatory cytokines by enhancing SIRT1 activation, thereby attenuating DR [5]. Additionally, a previous study demonstrated the treatment with the isoflavone formononetin in combination with induction of SIRT1 expression in kidney tissues of diabetic rats in therapeutically reducing the burden of oxidative stress [15]. Based on the aforementioned findings, we hypothesized that there is a potential regulatory relationship among CMS, SIRT1, and DR. To test this hypotheses, we investigated the effects of CMS on oxidative stress, inflammatory response, and cell apoptosis in the setting of DR in relation to activation of SIRT1.

## RESULTS

### SIRT1, lowly expressed in the STZ-induced rat DR model, can be rescued by CMS treatment

**Figure 1:**
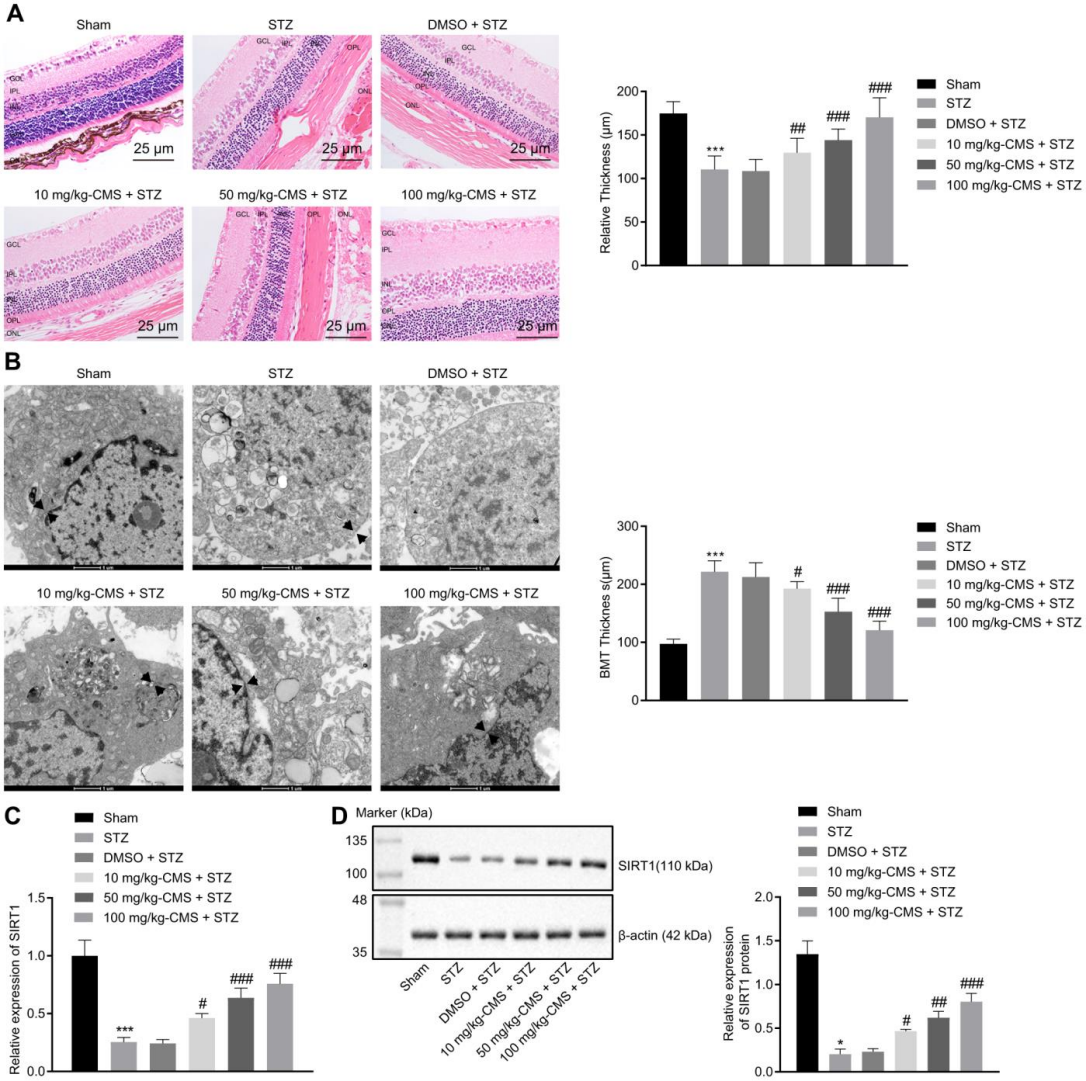
**SIRT1 was down-regulated in the STZ-induced DR rat model.** STZ-treated rats were further treated with DMSO, 10 mg/kg CMS, 50 mg/kg CMS, and 100 mg/kg CMS. n = 15 per treatment. (**A**) Representative images of retinal tissues observed by HE-staining (× 400) (scale bar = 25 μm) and quantitative analysis of thickness. * *p* < 0.05 compared to sham-operated rats and ^#^ *p* < 0.05 compared to STZ-treated rats. (**B**) Ultrastructure of rat retinal tissues observed under a TEM (× 10000) (scale bar = 1 μm). The arrow represents the capillary BMT, which is used to measure basement membrane width. (**C**) Expression pattern of SIRT1 measured by RT-qPCR in rat retinal tissues, normalized to β-actin. (**D**) Representative Western blots of SIRT1 protein and its quantitation in rat retinal tissues, normalized to β-actin. ^*^ *p* < 0.05, ^**^ *p* < 0.01, ^***^ *p* < 0.001, compared to the sham-operated rats, and ^#^ *p* < 0.05, ^##^ *p* < 0.01, ^###^ *p* < 0.001, compared to the rats injected with STZ and treated with DMSO. The results were measurement data and expressed as mean ± standard deviation. Comparisons between multiple groups were analyzed by one-way ANOVA with Tukey’s post hoc test. (n = 15). DR, diabetic retinopathy; STZ, streptozotocin; RT-qPCR, reverse transcription-quantitative polymerase chain reaction; SIRT1, Sirtuin 1; TEM, transmission electron microscopy; ANOVA, analysis of variance; n, number; GCL, ganglion cell layer; INL, inner nuclear layer; IPL, inner plexiform layer; ONL, outer nuclear layer; OPL, outer plexiform layer DMSO, dimethyl sulfoxide.

First, to study the effects of CMS on DR, we established the rat model of DR using an intraperitoneal injection of STZ, administered at a dose of 60 mg/kg. A blood glucose level over 16.7 mmol/L at 48 h was indicative of successful model establishment. The age-matched sham-operated rats were administrated with an equivalent same volume of sodium citrate before CMS treatment. Two weeks after STZ induction, the rats were treated with DMSO, 10 mg/kg CMS, 50 mg/kg CMS, and 100 mg/kg CMS. HE staining was subsequently conducted to observe the structural integrity of retinal tissues of rats following DR induction. This examination revealed that the vascular intima of retinal tissues in the sham-operated rats consisted of a layer of intact and continuous endothelial cells without hyperplasia. In comparison with the sham-operated rats, the STZ-treated rats exhibited obvious edema, capillary wall thickening, endothelial cell hyperplasia and fibrous tissue hyperplasia, all of which could be partially relieved by administering CMS in a dose-dependent manner (Figure 1A). Next, the ultrastructure of retinas was observed under a transmission electron microscope (TEM), which showed that, compared with the sham-operated rats, the BMT value in STZ-treated rats was increased, while treatment with CMS decreased the BMT values in a dose-dependent manner (Figure 1B). In addition, the results of RT-qPCR and Western blot analysis showed that, compared with the sham-operated rats, the expression of SIRT1 was notably decreased in STZ-treated rats, which was restored by CMS treatment in a dose-dependent manner (Figure 1C, 1D). Therefore, the expression of SIRT1 was inhibited in STZ rats, and CMS could elevate the expression of SIRT1 in STZ rats.

### CMS alleviated oxidative stress, inflammation and apoptosis in the retina of DR rats

**Figure 2:**
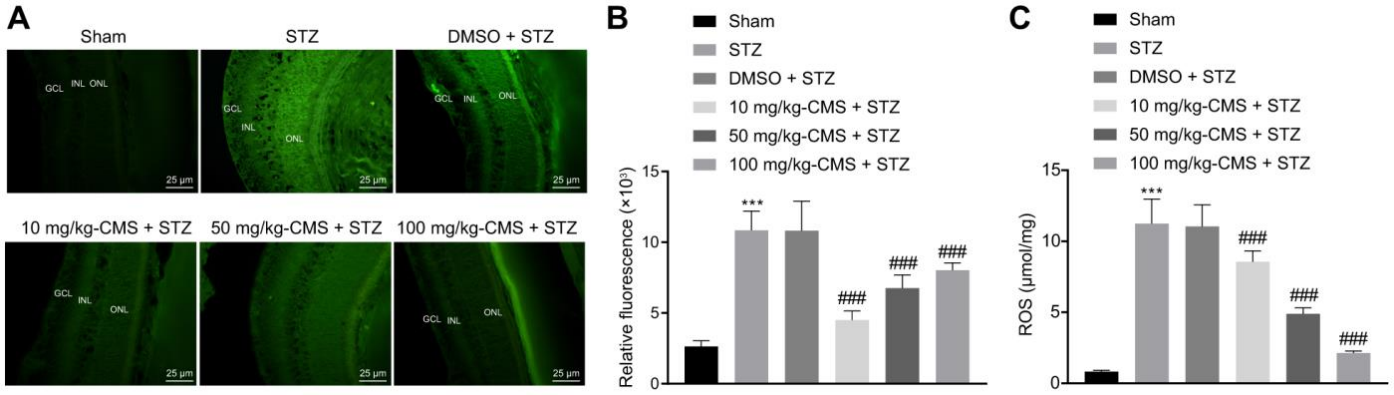
**CMS decreased the level of ROS in the retinal tissues of DR rats.** (**A**) Representative images of ROS shown by CM-H2DCFDA staining (green) in the retinal tissues of sham-operated and STZ-treated rats administrated with DMSO, 10 mg/kg CMS, 50 mg/kg CMS, and 100 mg/kg CMS (× 400) (scale bar = 25 μm). (**B**) Relative fluorescence in retinal tissues. (**C**) Quantitative analysis of ROS content in the retinal tissues. ^*^ *p* < 0.05, ^**^ *p* < 0.01, ^***^ *p* < 0.001, compared to the sham-operated rats and ^#^ *p* < 0.05, ^##^ *p* < 0.01, ^###^ *p* < 0.001, compared to the rats injected with STZ and treated with DMSO. Data were shown as mean ± standard deviation. Comparisons between multiple groups were analyzed by one-way ANOVA with Tukey’s post hoc test. (n = 15). CMS, coumestrol, DR, diabetic retinopathy; STZ, streptozotocin; ROS, reactive oxygen species; DMSO, dimethyl sulfoxide; ANOVA, analysis of variance; n, number.

**Figure 3:**
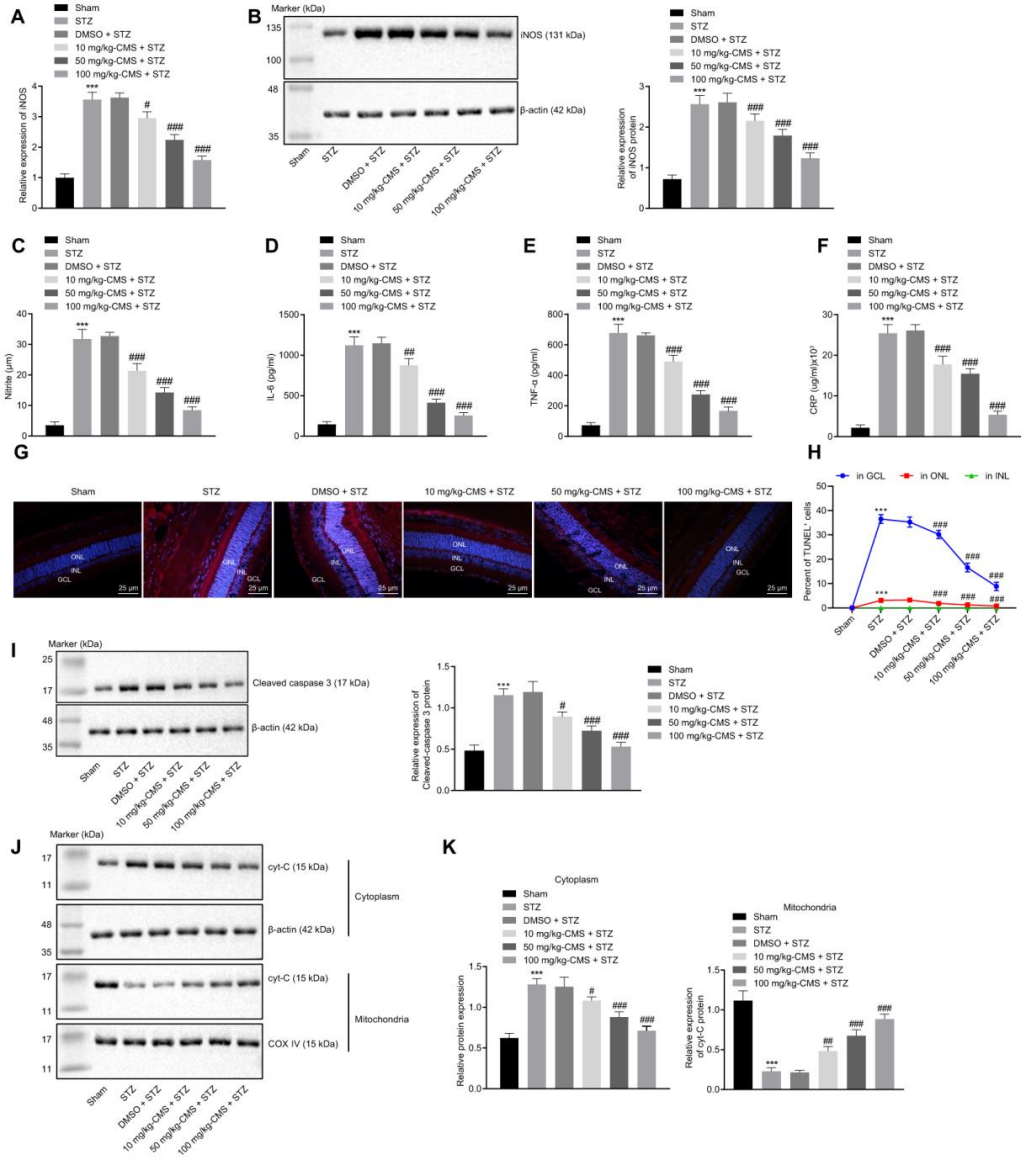
**CMS reduced oxidative stress, inflammation, and apoptosis in DR rats.** STZ-treated rats were treated with DMSO, 10 mg/kg CMS, 50 mg/kg CMS, and 100 mg/kg CMS. n = 15 per treatment. (**A**) Expression pattern of iNOS as determined by RT-qPCR in rat retinal tissues, normalized to β-actin. (**B**) Representative Western blots of iNOS protein and its quantitation in rat retinal tissues, normalized to β-actin. (**C**) Expression of NO measured by nitrite test in rat retinal tissues. (**D**–**F**) Expression patterns of IL-6 (**D**), TNF-α (**E**), and CRP (**F**) measured by ELISA in the cell supernatant. (**G**, **H**) Representative images of apoptotic cells (× 400) (scale bar = 25 μm) (**G**) and cell apoptosis in rat retinal tissues (**H**) by TUNEL. (**I**) Representative Western blots of cleaved caspase-3 protein and its quantitation in rat retinal tissues, normalized to β-actin. (**J**, **K**) Representative Western blots of Cyt-C protein and its quantitation in the cytoplasm and mitochondria, normalized to β-actin. ^*^ *p* < 0.05, ^**^ *p* < 0.01, ^***^ *p* < 0.001, compared to the sham-operated rats, and ^#^ *p* < 0.05, ^##^ *p* < 0.01, ^###^ *p* < 0.001, compared to the rats injected with STZ and treated with DMSO. The results were measurement data, which were expressed as mean ± standard deviation. Comparisons between multiple groups were analyzed by one-way ANOVA with Tukey’s *post hoc* test (n = 15). iNOS, inducible nitric oxide synthase; CMS, coumestrol, STZ, streptozotocin; DMSO, dimethyl sulfoxide; NO, nitric oxide; RT-qPCR, reverse transcription-quantitative polymerase chain reaction; IL-6, interleukin-6; TNF-α, tumor necrosis factor α; CRP, C-reactive protein; ELISA, Enzyme linked immunosorbent assay; GCL, ganglion cell layer; INL, inner nuclear layer; ONL, outer nuclear layer; Cyt-C, cytochrome c; ANOVA, analysis of variance; n, number.

Further, we examined the expression patterns of oxidative stress-related factors in the retinal tissues of STZ-treated rats using DCFDA and lipid peroxidation MDA assay kits. The results suggested that, compared with the sham-operated rats, the ROS and MDA levels were significantly increased, while the SOD level was decreased in the retinal tissues of rats injected with STZ, and all these changes were negated by treatment with CMS in a dose-dependent manner (Figure 2, Table 1). Additionally, the results of RT-qPCR and Western blot analysis suggested that, compared with the sham-operated rats, the expression of iNOS was notably increased in the retinal tissues of STZ-treated rats, while treatment with CMS inhibited the expression of iNOS induced by STZ in a dose-dependent manner (Figure 3A, 3B). Nitrite testing showed that, compared with the sham-operated rats, the expression of NO was notably increased in STZ-treated rats, which was reversed by CMS in a dose-dependent manner (Figure 3C). According to the results of ELISA, compared with the sham-operated rats, STZ-treated rats presented with notably increased contents of IL-6, TNF-α, and CRP in the cell supernatant. In contrast to the STZ-treated rats injected with DMSO, the STZ-treated rats injected with CMS presented with considerably reduced expression contents of IL-6, TNF-α, and CRP in the cell supernatant, while the anti-inflammatory effect of CMS was dose-dependent (Figure 3D–3F). Cell apoptosis detected by TUNEL exhibited an increase in cell apoptosis in STZ-treated rats, while subsequent treatment with CMS resulted in dose-dependent reductions in cell apoptosis in STZ-treated rats (Figure 3G, 3H). Additionally, the Western blot analysis showed that the protein expression of apoptosis-related factor cleaved caspase-3 was higher in the retinal tissues of STZ-treated rats relative to the sham-operated rats, which could be reduced by CMS treatment in a dose-dependent manner (Figure 3I). As shown in Figure 3J, 3K, the cytoplasmic Cyt-C protein expression was elevated in the retinal tissues of STZ-treated rats, which was decreased upon CMS treatment. On the other hand, opposite changes in Cyt-C expression were observed in the mitochondria. Taken together, the aforementioned findings exhibited that CMS was effective in alleviating the oxidative stress, inflammation and cell apoptosis in the retina of the STZ rat model.

**Table 1:** CMS reduced the expression of MDA while increasing that of SOD in retinal tissues of STZ-treated rats.

**Group**	**SOD (U/mg)**	**MDA (umol/mg)**
sham	23.15 ± 1.85	2.57 ± 0.28
STZ	8.37 ± 0.94^*^	7.29 ± 0.64^*^
STZ + DMSO	8.31 ± 0.89	7.25 ± 0.68
10 mg/kg-CMS + STZ	12.36 ± 1.18^#^	6.23 ± 0.71^#^
50 mg/kg-CMS + STZ	17.25 ± 1.64^#^	5.03 ± 0.38^#^
100 mg/kg-CMS + STZ	19.37 ± 1.85^#^	4.12 ± 0.51^#^

Notes: MDA level was detected using lipid peroxidation MDA assay and SOD was detected by WST-8 SOD assay. ^*^ *p* < 0.05 compared to the sham-operated rats, and ^#^ *p* < 0.05 compared to the rats injected with STZ + DMSO. Data were shown as mean ± standard deviation and compared using one-way ANOVA with Tukey’s post hoc test. n = 15 for rats upon each treatment. CMS, coumestrol; MDA, malondialdehyde; SOD, superoxide dismutase; STZ, streptozotocin; DMSO, dimethyl sulfoxide.

### CMS activated SIRT1 expression in HG-exposed hRMECs

**Figure 4:**
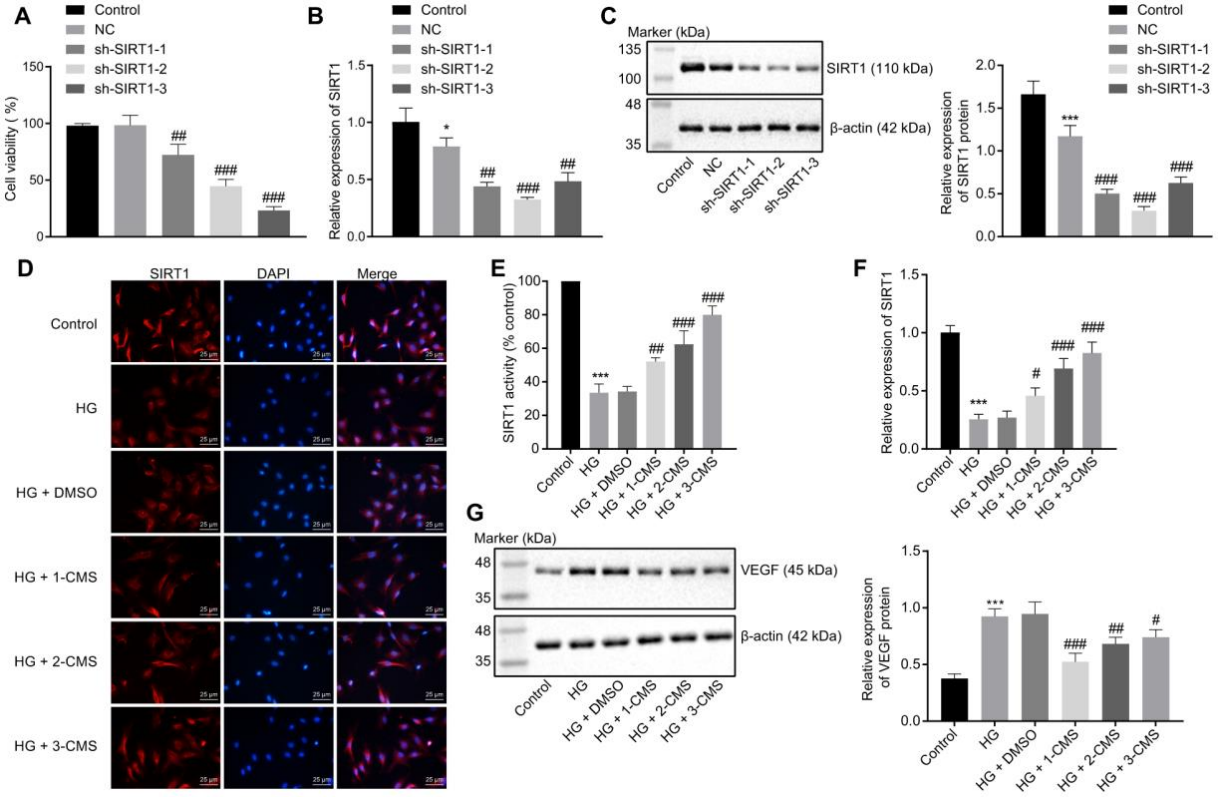
**CMS activated the expression of SIRT1 in HG-treated hRMECs.** hRMECs were transfected with sh-SIRT1-1, sh-SIRT1-2, or sh-SIRT1-3, and HG-exposed hRMECs were treated with DMSO, 1-CMS, 2-CMS, or 3-CMS, respectively. (**A**) Cell viability assessed by CCK-8 assay. (**B**) SIRT1 expression pattern determined by RT-qPCR in hRMECs, normalized to β-actin. (**C**) Representative Western blots of SIRT1 protein and its quantitation in hRMECs, normalized to β-actin. (**D**, **E**) Representative images (× 400) (scale bar = 25 μm) (**D**) as well as SIRT1 activity and nuclear accumulation in hRMECs (**E**) detected by immunofluorescence staining. (**F**) Expression pattern of SIRT1 as determined by RT-qPCR in hRMECs, normalized to β-actin. (**G**) Representative Western blots of VEGF protein and its quantitation in hRMECs, normalized to β-actin. ^*^ *p* < 0.05, ^**^ *p* < 0.01, ^***^ *p* < 0.001, compared to the control cells, and ^#^ *p* < 0.05, ^##^ *p* < 0.01, ^###^ *p* < 0.001, compared to cells stimulated with NC or HG + DMSO. The results were measurement data and expressed as mean ± standard deviation. Comparisons between multiple groups were analyzed by one-way ANOVA with Tukey’s post hoc test. The cell experiments were repeated three times independently. NC, negative control; CMS, coumestrol, HG, high glucose; DMSO, dimethyl sulfoxide; SIRT1, sirtuin 1; RT-qPCR, reverse transcription-quantitative polymerase chain reaction; VEGF, vascular endothelial growth factor; ANOVA, analysis of variance.

After establishing that that CMS could increase SIRT1 expression in STZ-treated rats, we next aimed to investigate the interaction between CMS and SIRT1 in hRMECs under HG conditions by conducting SIRT1 loss-of-function and rescue experiments. The viability of hRMECs was decreased at 48 h following transfection with sh-SIRT1-1, sh-SIRT1-2, or sh-SIRT1-3 (Figure 4A). The results of RT-qPCR and Western blot analysis demonstrated that the expression of SIRT1 was diminished in hRMECs transfected with sh-SIRT1-1, sh-SIRT1-2, or sh-SIRT1-3 (Figure 4B, 4C), where sh-SIRT1-2 exhibited the strongest silencing effect, and was therefore selected for subsequent experimentation. Immunofluorescence staining revealed that stimulation with HG decreased the activity and nuclear accumulation of SIRT1 in hRMECs; however, CMS treatment resulted in dose-dependent increases of SIRT1 in HG-exposed hRMECs, with 3-CMS showing a more pronounced elevation (Figure 4D, 4E). According to the results of RT-qPCR, the expression of SIRT1 was decreased in hRMECs stimulated with HG, while CMS treatment led to dose-dependent increases in the expression of SIRT1 in HG-exposed hRMECs, with the highest SIRT1 expression evident upon 3-CMS treatment (Figure 4F). Hence, 3-CMS was selected for subsequent experimentation. Meanwhile, we tested the effect of CMS on the expression of VEGF protein by performing Western blot analysis, which showed increased VEGF protein level in HG-treated hRMECs, whereas further CMS treatment provoked a dose-dependent decrease in the VEGF protein level (Figure 4G). Thus, from the preceding, we find that CMS exhibited the potential to enhance the SIRT1 expression while downregulating the VEGF expression in HG-exposed hRMECs.

### CMS inhibited oxidative stress and inflammation by activating SIRT1 in HG-treated hRMECs

**Figure 5:**
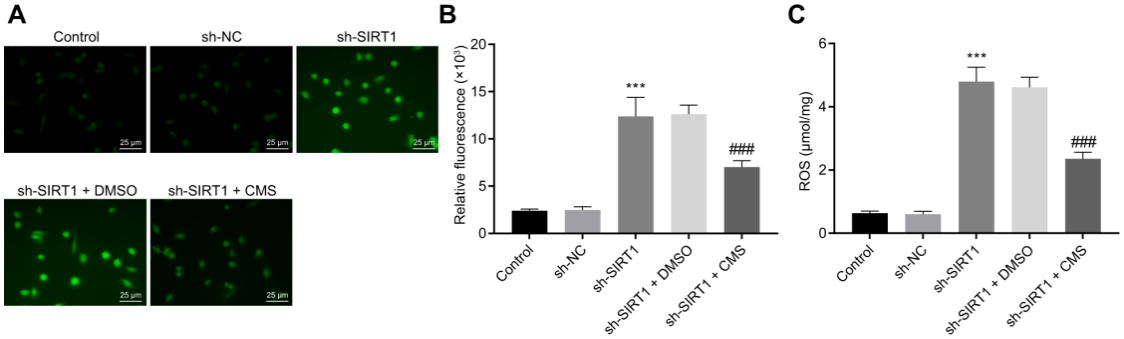
**CMS decreased the level of ROS in HG-treated hRMECs.** (**A**) Representative images of ROS shown by CM-H2DCFDA staining (green) in hRMECs treated with sh-NC, sh-SIRT1, sh-SIRT1 + DMSO and sh-SIRT1 + CMS, respectively (× 400) (scale bar = 25 μm). (**B**) Relative fluorescence in the hRMECs. (**C**) Quantitative analysis of ROS content in hRMECs. ^*^ *p* < 0.05, ^**^ *p* < 0.01, ^***^ *p* < 0.001, compared to sh-NC-treated hRMECs and ^#^ *p* < 0.05, ^##^ *p* < 0.01, ^###^ *p* < 0.001, compared to the hRMECs treated with sh-SIRT1 + DMSO. The results were measurement data and expressed as mean ± standard deviation. Comparisons between multiple groups were analyzed by one-way ANOVA with Tukey’s post hoc test. The cell experiments were repeated three times independently. CMS, coumestrol, ROS, reactive oxygen species; hRMECs, human retinal microvascular endothelial cells; SIRT1, sirtuin 1; DMSO, dimethyl sulfoxide; ANOVA, analysis of variance.

**Figure 6:**
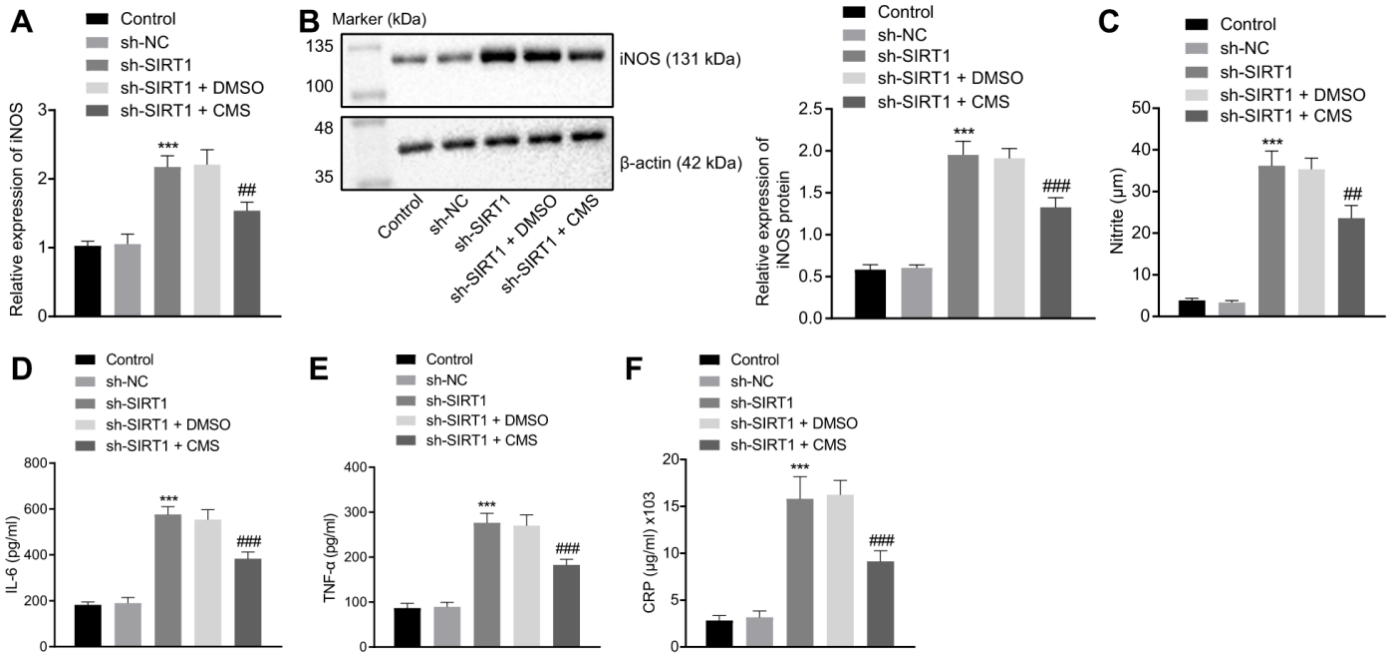
**CMS suppressed HG-induced oxidative stress and inflammation in hRMECs through activating SIRT1.** hRMECs were treated with sh-NC, sh-SIRT1, sh-SIRT1 + DMSO and sh-SIRT1 + CMS, respectively. (**A**) Expression pattern of iNOS as determined by RT-qPCR in hRMECs, normalized to β-actin. (**B**) Representative Western blots of iNOS protein and its quantitation in hRMECs, normalized to β-actin. (**C**), Expression pattern of NO as determined by nitrite test in hRMECs. (**D**–**F**), Expression patterns of IL-6 (**D**), TNF-α (**E**), and CRP (**F**) as measured by ELISA in the cell supernatant. ^*^ *p* < 0.05, ^**^ *p* < 0.01, ^***^ *p* < 0.001, compared to sh-NC-treated cells, and ^#^ *p* < 0.05, ^##^ *p* < 0.01, ^###^ *p* < 0.001, compared to cells stimulated with sh-SIRT1 and treated with DMSO. The results were measurement data and expressed as mean ± standard deviation. Comparisons between multiple groups were analyzed by one-way ANOVA with Tukey’s *post hoc* test. The cell experiments were repeated three times independently. NC, negative control; CMS, coumestrol, HG, high glucose; hRMECs, human retinal microvascular endothelial cells; SIRT1, sirtuin 1; DMSO, dimethyl sulfoxide; RT-qPCR, reverse transcription-quantitative polymerase chain reaction; IL-6, interleukin-6; TNF-α, tumor necrosis factor α; CRP, C-reactive protein; ELISA, Enzyme linked immunosorbent assay; ANOVA, analysis of variance.

To evaluate whether CMS regulated oxidative stress and inflammation through SIRT1, we silenced the SIRT1 expression in hRMECs. The results of DCFDA and lipid peroxidation MDA assays illustrated that the levels of ROS and MDA were augmented, while the activity of SOD was diminished by SIRT1 silencing in hRMECs. However, CMS reversed the effects of sh-SIRT1 on the expression pattern of oxidative stress-related factors in hRMECs (Figure 5, Table 2). In addition, the results of RT-qPCR and Western blot analysis demonstrated that the iNOS expression was elevated by SIRT1 silencing in hRMECs, however this increase could be reduced by CMS treatment (Figure 6A, 6B). The nitrite test revealed that the expression of NO was increased in hRMECs in the absence of SIRT1, but that this effect was reversed by CMS treatment (Figure 6C). According to the results of ELISA, hRMECs had increasing concentrations of IL-6, TNF-α, and CRP upon SIRT1 knockdown, while concomitant CMS treatment reversed these elevations of inflammatory markers (Figure 6D–6F). Therefore, CMS was effective in alleviating the oxidative stress and inflammation in hRMECs through activating SIRT1.

**Table 2:** CMS reduced the expression of MDA while increasing that of SOD in hRMECs by upregulating SIRT1.

**Group**	**SOD (U/mg)**	**MDA (umol/mg)**
control	26.74 ± 2.38	2.24 ± 0.17
sh-NC	26.9 ± 2.482	2.02 ± 0.22
sh-SIRT1	7.25 ± 0.68^*^	15.37 ± 1.24^*^
sh-SIRE1 + DMSO	7.34 ± 0.32	15.28 ± 1.12
sh-SIRT1 + CMS	15.38 ± 1.44^&^	8.79 ± 0.76^&^

Notes: MDA level was detected using lipid peroxidation MDA assay and SOD was detected by WST-8 SOD assay. ^*^ *p* < 0.05 compared to cells treated with sh-NC, and ^#^ *p* < 0.05 compared to cells treated with sh-SIRE1 + DMSO. Data were shown as mean ± standard deviation, and compared using one-way ANOVA with Tukey’s post hoc test. The cell experiment was repeated independently three times. CMS, coumestrol; MDA, malondialdehyde; SOD, superoxide dismutase; hRMECs, human retina microvascular endothelial cells; sh-NC, short hairpin RNA-negative control; DMSO, dimethyl sulfoxide; SIRT1, sirtuin 1.

### CMS inhibits apoptosis of HG-treated hRMECs

**Figure 7:**
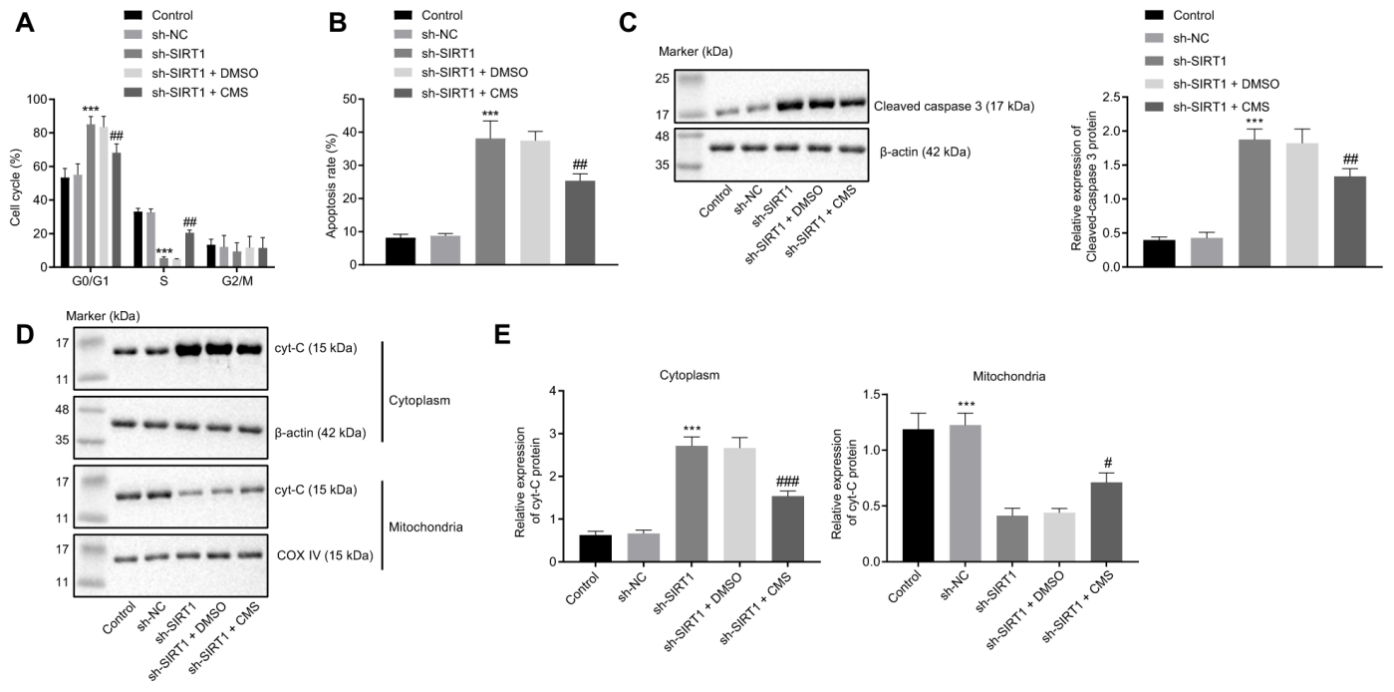
**CMS suppressed the apoptosis of HG-treated hRMECs**. hRMECs were treated with sh-NC, sh-SIRT1, sh-SIRT1 + DMSO and sh-SIRT1 + CMS, respectively. (**A**) Cell cycle distribution as determined by flow cytometry analysis. (**B**) Cell apoptosis as determined by flow cytometry analysis. (**C**) Representative Western blots of cleaved caspase-3 protein and its quantitation in hRMECs, normalized to β-actin. (**D**, **E**) Western blots of Cyt-C protein (**D**) and its quantitation (**E**) in the cytoplasm and mitochondria, normalized to β-actin. ^*^ *p* < 0.05, ^**^ *p* < 0.01, ^***^ *p* < 0.001, compared to sh-NC-treated cells, and ^#^ *p* < 0.05, ^##^ *p* < 0.01, ^###^ *p* < 0.001, compared to cells stimulated with sh-SIRT1 + DMSO. The results were the measurement data and expressed as mean ± standard deviation. Comparisons between multiple groups should be analyzed by one-way ANOVA with Tukey's p*ost hoc* test. The cell experiments were repeated three times independently. CMS, coumestrol; HG, high glucose; hRMECs, human retinal microvascular endothelial cells; NC, negative control; DMSO, dimethyl sulfoxide; SIRT1, sirtuin 1; NO, nitric oxide; Cyt-C, cytochrome c; ANOVA, analysis of variance.

At last, to illustrate whether CMS mediated SIRT1 in the process of cell apoptosis, we evaluated cell apoptosis and assessed the expression patterns of several apoptosis-related factors in hRMECs after CMS treatment and/or SIRT1 knockdown. Flow cytometry showed that the apoptosis rate of hRMECs was increased upon SIRT1 knockdown, which could be reversed by CMS treatment (Figure 7A, 7B). Furthermore, Western blot analysis showed that hRMECs treated with sh-SIRT1 exhibited a higher expression of cleaved caspase-3, which could be diminished by CMS treatment (Figure 7C). Additionally, Cyt-C protein expression was amplified in the cytoplasm of hRMECs treated with sh-SIRT1, but this enhancement was impeded by CMS treatment (Figure 7D, 7E). On the whole, these data suggested that CMS could inhibit the apoptosis of hRMECs by activating the expression of SIRT1.

## DISCUSSION

DR is a progressive ophthalmopathy as a consequence of long-term accumulated pathological alterations provoked by chronic hyperglycemia in the retina of diabetic patients [6]. Several treatment modalities, such as physical exercise and drug therapy have been demonstrated to be effective in alleviating the progression of DR [16]. Findings of one intervention study implied phytoestrogens such as biochanin A would be effective in DR treatment [17]. By establishing a STZ-induced DR model in rats, we investigated the potential roles of CMS and SIRT1 in oxidative stress, inflammatory response, and cell apoptosis in the current study, and later elucidated that CMS induced the activation of SIRT1 and effectively constrained oxidative stress, inflammatory response, as well as cell apoptosis in rats with DR.

Our initial findings demonstrated that SIRT1 was expressed at a low level in the retinal tissues of rats with STZ-induced DR. SIRT1, nuclear protein, is able to regulate inflammation and apoptosis along with various other metabolic pathways through deacetylation of histones, non-histones, and transcription factors [18]. In consistency with our findings, Li et al. demonstrated that SIRT1 was down-regulated in endothelial cells of STZ-induced diabetic mice [19]. Additionally, activation of retinal SIRT1 exhibited protective properties against retinal damage in the diabetic milieu [5].

Next, we investigated the relationship between CMS and SIRT1 in rats with DR, finding that CMS could stimulate SIRT1 expression, thereby demonstrating an association between the functions of CMS on DR through SIRT1. Furthermore, our findings revealed that CMS inhibited oxidative stress and inflammatory response in rats with DR by activating SIRT1, as indicated by the decreased levels of ROS, MDA, iNOS, NO, and other inflammatory factors as well as an increased activity of SOD. Induction of ROS production a resultant mitochondrial dysfunction is a major causative factor of retinal cell death in DR [20]. DR has also been regarded as a stimulator for the release of pro-inflammatory cytokines including IL-1β, IL-6 and TNF-α [21, 22]. A previous study demonstrated that CMS inhibited the production of hydrogen peroxide-induced ROS, lipid peroxidation, and further averted the reduction of intracellular glutathione levels and SOD activity [23]. Moreover, CMS has exhibited effectiveness as a preventive modality against IL-1β-induced catabolic effects by inhibiting inflammation and associated inflammatory cytokines in chondrocytes [24]. Oxidative stress has been regarded as the common denominator of key pathways involved in the progression and development of DR [25]. Inhibition of SIRT1 by overexpression of miR-138 results in increased ROS levels in the HG-induced retinal pigment epithelial cell line ARPE-19 [26]. Moreover, observations of mitigated oxidative stress, such as reduced content of MDA, have revealed a link with the activation of SIRT1 in the renal tissues of STZ-induced diabetic rats [27]. Furthermore, elevated SIRT1 expression is associated with reduced oxidative stress, which consequently ameliorates DR pathology [18]. In addition, activated SIRT1 has the capacity to down-regulate several pro-inflammatory cytokines, and inhibit oxidative stress and cell inflammation, thereby improving DR [28]. These findings are consistent with the inhibitory role of CMS-mediated SIRT1 activation in oxidative stress and inflammation in the context of DR, as validated in this study.

Furthermore, our findings demonstrated that CMS suppressed the apoptosis of hRMECs, thereby ameliorating DR through activation of SIRT1, as supported by the reduced levels of Cyt-C, and caspase-3. Phytoestrogens, including CMS, genistein, and daidzein, were reported to suppress apoptosis by acting synergistically with 17β-estradiol [29]. For example, genistein, inhibited chemical hypoxia-induced cell apoptosis by suppressing the mitochondrial apoptotic pathway and the level of caspase-3 [30]. Additionally, activated SIRT1 in combination with the silenced protein arginine methyltransferases demonstrated the capacity to suppress the oxidative stress-induced apoptosis of retinal pigment epithelial cells in DR, as evidenced by a reduced level of caspase-3 [31]. Moreover, another study suggested that activation of SIRT1 was related to inhibited HG-induced apoptosis and inflammation cytokine production in DR [32]. Together with this background, our present findings suggested that CMS might be an important substance for DR treatment, delaying the progression of DR by activating SIRT1.

In summary, the key findings from this study supported our hypothesis that CMS could inhibit oxidative stress, inflammatory response, and cell apoptosis in rats with DR by activation of SIRT1. This finding may inspire potentially important therapeutic implications in the treatment of oxidative stress and inflammatory response in patients suffering from DR.

## MATERIALS AND METHODS

### Ethics statement

The study was in strict accordance with the guidelines of association for research in vision and ophthalmology (ARVO) for the use of animals. Optimal measures were taken to minimize the number of animals as well as their suffering.

### STZ-induced rat DR model establishment

A total of 80 specific pathogen-free (SPF) grade male Sprague-Dawley (SD) rats weighing 250 - 300 g were purchased from Hunan SJA Laboratory Animal Co., Ltd. (Changsha, China). Among the mice, 15 sham-operated rats were fed a normal diet while another 60 rats were fed a high-fat diet. After a 12 h period of fasting, the rats on a high-fat diet were administered an intraperitoneal injection with STZ dissolved using 0.1 mol/L citrate buffer (pH = 4.5) at a dose of 60 mg/kg. After 72 h, blood samples were harvested from the tail vein and the blood glucose level was measured. The sham-operated rats were injected with an equal dose of citric acid buffer and the blood glucose was measured using the off-line blood glucose monitoring system (Glucotrend-2; Roche Diagnostics GmbH, Mannheim, Germany). A blood glucose level exceeding 16.7 mmol/L for a week was indicative of successful DR model establishment [33, 34]. The STZ-treated rats were administered an intraperitoneal injection with an equal volume of dimethyl sulfoxide (DMSO) or CMS. In brief, 100% DMSO was added to dilute the CMS, and from the day of model establishment by STZ, the STZ-treated rats were subcutaneously injected with DMSO (n = 15), 10 mg/kg (n = 15), 50 mg/kg (n = 15) and 100 mg/kg (n = 15) CMS for 8 weeks, respectively. The rats in the sham and STZ groups were administered with equal volumes of normal saline.

### Cell culture and treatment

Human retinal microvascular endothelial cells (hRMECs) were purchased from the Beijing Beichuang Institute of Biotechnology (Beijing, China) and cultured using Dulbecco’s modified Eagle’s medium (DMEM; Gibco, Carlsbad, CA, USA) containing 10% fetal bovine serum (FBS; Gibco), 100 U/mL penicillin and 100 μg/mL streptomycin in an incubator at 37° C with 5% CO_2_ saturation. As hyperglycemia and hyperlipidemia are characteristic of diabetes, hRMECs were cultured using HG (70 nM) to induce DR cell models. In brief, the cells were inoculated in a 6-cm medium at a density of 2 × 10^5^ and incubated at 37° C with 5% CO_2_ until the cell confluence reached 70 - 80%. On the day before transfection, the cells were trypsinized and then dispersed into a single cell suspension with the culture medium. Next, the single cell suspension was inoculated in a 6-well plate at a density of 2 × 10^5^ cells per well. Subsequently, 10 μL of shRNA against SIRT1 (sh-SIRT1) was mixed with the Opti-minimum essential medium (MEM) to a total volume of 250 μL. After mixing for 5 min at room temperature, the samples were incubated with an equal volume of liposome for 20 min at room temperature to formulate an shRNA-liposome complex. Next, the complex was transferred to a 6-well plate at a density of 0.5 nL per well and mixed gently, whereupon 1.5 mL of serum-free DMEM was added to each well and cultured at 37° C with 5% CO_2_ conditions for 6 h. Next, the medium was replaced with DMEM containing normal serum and maintained in an incubator with 5% CO_2_ at 37° C.

### Cell counting kit-8 (CCK-8) assay

The viability of hRMECs was assessed using the CCK-8 kit (Dojindo Molecular Technologies, Gaithersburg, MD, USA) upon SIRT1 knockdown. Briefly, the hRMECs were cultured in 96-well plates. After transfection with the shRNA plasmids for 48 h, the cells were incubated with 10 μL of CCK-8 solution for 1 h in a 37° C incubator with 5% CO_2_ and 95% relative humidity. A microplate reader (Bio-Rad, Hercules, CA, USA) was employed to record the absorbance value at the wavelength of 450 nm.

### Measurement of reactive oxygen species (ROS), 3,4-methylenedioxyamphetamine (MDA) and superoxide dismutase (SOD) levels

The production of ROS in cells was measured using the CM-H2DCFDA kit (#C6827, Thermo Fisher Scientific, Rockford, IL, USA). The MDA and SOD were detected using the lipid peroxidation MDA assay kit (#S0131) and WST-8 SOD assay kit (#S010) in strict accordance with the provided instructions. The corresponding value was expressed as the percentage of average absorbance normalized to that of normal control absorbance.

### Nitrite test

Nitric oxide (NO) is unstable, readily forming nitrates and nitrites in cells. Therefore, the nitrite content in the supernatant was measured as a marker of NO production using an oxidized nitrite kit (Beyotime Institute of Biotechnology, Jiangsu, China). In brief, according to the provided instructions, the supernatant was sequentially mixed with equal volumes of Gris reagent I and Gris reagent II, and the concentration of nitrites was calculated according to the optical density (OD) value at the wavelength of 540 nm measured using the smart-spec plus spectrophotometer (Bio Rad laboratories, Hercules, CA, USA) [35].

### Enzyme linked immunosorbent assay (ELISA)

The levels of interleukin-6 (IL-6), tumor necrosis factor α (TNF-α), and C-reactive protein (CRP) in the cell supernatant were detected by means of ELISA kits (R & D Systems, Minneapolis, Minnesota, USA) according to the provided instructions using the smart spec-plus spectrophotometer (Bio Rad laboratories, Hercules, CA, USA) [36].

### Hematoxylin-eosin (HE) staining

After ophthalmectomy, the rat eyeballs were fixed by immersion in 4% paraformaldehyde for 48 h. The retina and sclera were dehydrated using gradient ethanol and embedded with paraffin. Next, the paraffin-embedded sections were dewaxed with xylene and rinsed successively with ethanol of declining concentration (99.9%, 97%, 75%, 50%) and distilled water. Then sections were stained with hematoxylin for 1 - 3 min, rinsed under running water for 1 min after each step, differentiated using 1% hydrochloric acid alcohol for 20 s, and rinsed in phosphate-buffered saline (PBS) for 30 s. Next, the sections were stained with eosin for 1 min, dehydrated with gradient ethanol, cleaned using xylene, and then finally sealed with Histomount. The retinas of rats in each group were observed and photographed under a high-power optical microscope (Olympus Optical Co., Ltd, Tokyo, Japan).

### Transmission electron microscopy (TEM)

The anterior segment of rats was removed immediately after the ophthalmectomy and immersed in 2.5% glutaraldehyde solution to facilitate retina separation. The retinas were fixed with 4% glutaraldehyde for 1 h and then rinsed with PBS for 2 h. Next, retinas were fixed with 1% osmic acid, dehydrated using ethanol, embedded with Epon epoxy resin, followed by staining for TEM with uranium acetate and lead citrate (each for 15 min), and cut into ultrathin sections. Retina samples were then observed and photographed under a TEM (HT7700; Exalens, Hitachi Plant Technologies Ltd, Tokyo, Japan). With the help of a 20-spoke grid over each capillary micrograph, the thickness of the basement membrane at each point of intersection with a spoke was measured. At least 10 random views were selected of the retinal capillaries extending from the outer plexiform and ganglion cell layers from each rat to measure the basement membrane thickness (BMT) of capillaries. Finally, the mean BMT value was calculated from the four retinas.

### Terminal deoxynucleotidyl transferase-mediated dUTP-biotin nick end labeling (TUNEL) staining

Frozen rat retina sections were used for determination of DNA fragments of apoptotic cells. TUNEL staining was performed according to the provided instructions of the *in situ* cell death detection kit (KeyGEN BioTech Co., Ltd., Nanjing, China). Briefly, the retina sections were covered with protease K at 37° C for 20 min and then incubated with the TDT enzyme solution (45 μL balanced buffer solution containing 1.0 μL biotin-11-dUTP and 4.0 μL TdT enzyme) at 37° C for 60 min. Thereafter, the sections were incubated with streptavidin-TRITC at 37° C for 30 min, rinsed with PBS, and stained with DAPI at room temperature for 15 min. The sections were observed under a confocal laser microscope (Leica, Wetzlar Germany). The number of cells with broken DNA strands (TUNEL-positive cells) was calculated in five randomly selected fields of the retinal ganglion cell layer (GCL), inner nuclear layer (INL) and outer nuclear layer (ONL).

### Flow cytometry

Cells in the logarithmic growth phase (1 × 10^6^) were collected for cell cycle detection. In brief, the cells were fixed with 70% cold ethanol, and stained using 1 mL propidium iodide (PI) dye (50 μg/mL; Becton Dickinson Biosciences, Mountain View, CA, USA) in dark conditions for 30 min. The cell cycle was detected by means of fluorescein isothiocyanate (FITC) Calibur flow cytometry (Becton Dickinson Biosciences, Mountain View, CA, USA). Then, an equivalent amount of cells in the logarithmic growth phase were collected for apoptosis detection. The cells were suspended using 1 × Annexin buffer and stained with 5 μL Annexin-V-FITC (Becton Dickinson Biosciences, Mountain View, CA, USA) at room temperature for 10 min. Then, the cells were suspended using 300 μL of 1 × Annexin. The apoptosis rate was detected by means of flow cytometry.

### Immunofluorescence staining

The frozen sections of rat retina were rinsed, permeabilized with Triton-X and blocked using the goat serum blocking solution for 30 min. The sections were incubated with the rabbit anti-rat SIRT1 polyclonal antibody (ab189494, 1:100; Abcam, Cambridge, UK) overnight, respectively. Next, sections were rinsed several times with PBST, probed with the Alexa fluorescence-coupled secondary antibody (1:400) at room temperature for 3 h, stained by DAPI and sealed with glycerin. The sections were observed under a confocal microscope to obtain the staining images, which were then analyzed using the confocal software.

### Reverse transcription-quantitative polymerase chain reaction (RT-qPCR)

The total RNA content was extracted from rat retinas or hRMECs using the TRIzol reagents (Invitrogen, Carlsbad, CA, USA) and reversely transcribed into complementary DNA (cDNA) according to the provided instructions of TaqMan MicroRNA Assays Reverse Transcription Primer (4427975; Applied Bio-systems, Foster City, CA, USA). The conditions of the reverse transcription reaction were 37° C for 30 min and 85° C for 5 s. Then, 5 μL of the cDNA obtained was used as template for quantitative polymerase chain reaction (PCR) amplification using the QuantiTect SYBR Green RT-PCR kit. The reaction conditions were as follows: pre-denaturation at 95° C for 5 min, 45 cycles of denaturation at 95° C for 20 s, annealing at 60° C for 1 min, and extension at 72° C for 30 s. Real-time quantitative PCR results were analyzed based on the 2-ΔΔct theory. β-actin served as an internal reference. The fold changes were calculated based on relative quantification (2-ΔΔCt method) [37]. The primers are depicted in Supplementary Table 1.

### Western blot analysis

The total protein content was extracted from rat retinas or hRMECs using the radioimmunoprecipitation assay (RIPA) lysis buffer (Sigma-Aldrich, St. Louis, MO, USA). After centrifugation, the protein samples were isolated from the supernatant. The mitochondrial and cytoplasmic proteins of the cultured cells were extracted in compliance with the provided instructions of the mitochondrial separation kit (Thermo Fisher Scientific, Waltham, MA, USA) [38], followed by quantification by Bradford assay. Next, 40 μg of protein was subjected to separation by means of 10% sodium dodecyl sulfate-polyacrylamide gel electrophoresis (SDS-PAGE) and then electro-transferred onto a nitrocellulose membrane. A membrane blockade was conducted with 5% skimmed milk in Tris-buffered saline-Tween 20 (TBST) buffer at room temperature for 1 h, and then probed with the respective primary antibodies against SIRT1 (ab220807, 1:1000, Abcam), β-actin (PA1-183, 1:2000, Invitrogen), inducible nitric oxide synthase (iNOS) (PA3-030A; 1:2000, Invitrogen), Cytochrome C (Cyt-C; ab90529, 1:1000, Abcam), cytochrome oxidase (COX) IV (Bioworld Technology, St. Louis, MN, USA), cleaved caspase-3 (ab13847, 1:500, Abcam), glyceraldehyde-3-phosphate dehydrogenase (GAPDH; ab9485, 1:2500, Abcam), and vascular endothelial growth factor (VEGF; #PA5-16754, 1:1000, Thermo Fisher) at 4° C overnight. Subsequently, horseradish peroxidase (HRP)-conjugated secondary antibody goat anti-rabbit IgG (656120, 1:4000, Invitrogen, Carlsbad, CA, USA) was added and incubated with the membranes at room temperature for 1 h. β-actin served as an internal reference. Enhanced chemiluminescence reagent (ECL; Amersham Pharmacia Biotech, Piscataway, NJ, USA) was employed to visualize the immunocomplexes on the membrane and the Image Pro Plus 7.0 software (Media Cybernetics, Inc., Rockville, MD, USA) was used to quantify the band intensities.

### Statistical analysis

All data were processed using the SPSS 21.0 statistical software (IBM Corp., Armonk, NY, USA). Measurement data were summarized as mean ± standard deviation. The comparison between multiple groups was performed using one-way analysis of variance (ANOVA) with Tukey’s post hoc test. Statistical significance was assumed when *p* < 0.05.

## Supplementary Material

Supplementary Table 1
