# Supplementary material for: Coumestrol mitigates retinal cell inflammation, apoptosis, and oxidative stress in a rat model of diabetic retinopathy via activation of SIRT1
Source: Aging (Albany NY). 2021 Feb 1;13(4):5342–57. doi: 10.18632/aging.202467 (PMC7950241; doi:10.18632/aging.202467)
Supplement: Supplementary Table 1 [file aging-13-202467-s001.pdf]

SUPPLEMENTARY TABLE

Supplementary Table 1. Primer sequences for RT-qPCR.

Gene	Primer sequence
Rat β-actin	F: 5′-TCCTCCTGAGCGCAAGTACTCT-3'
	R: 5′-GCTCAGTAACAGTCCGCCTAGAA-3'
Rat SIRT1	F: 5′-CGCCTTATCCTCTAGTTCCTGTG-3'
	R: 5′-CGGTCTGTCAGCATCATCTTCC-3'
Rat iNOS	F: 5'-ATTCAGATCCCGAAACGC-3'
	R: 5′-CCAGAACCTCCAGGCACA-3'
Human β-actin	F: 5′-TGCTCGACAACGGCTCCGGCATGT-3'
	R: 5′-CCAGCCAGGTCCAGACGCAGGAT-3'
Human SIRT1	F: 5′-GAAAATGCTGGCCTAATAGACTTG-3'
	R: 5′-TGGTACAAACAAGTATTGATTACCG-3'
Human iNOS	F: 5'-TCACGACACCCTTCACCACAA-3'
	R: 5′-CCATCCTCCTGCCCACTTCCTC-3'

Notes: RT-qPCR, reverse transcription-quantitative polymerase chain reaction; SIRT1, sirtuin 1; iNOS, inducible nitric oxide synthase; F, forward; R, reverse.
